# Comparison of cine cardiac magnetic resonance and echocardiography derived diameters of the aortic root in a large population-based cohort

**DOI:** 10.1038/s41598-022-19461-5

**Published:** 2022-09-12

**Authors:** Jan-Per Wenzel, Julius Nikorowitsch, Ramona bei der Kellen, Luisa Dohm, Evaldas Girdauskas, Gunnar Lund, Peter Bannas, Stefan Blankenberg, Tilo Kölbel, Ersin Cavus, Kai Müllerleile, Michael Gerhard Kaul, Gerhard Adam, Julius Matthias Weinrich

**Affiliations:** 1 Department of Cardiology, University Heart and Vascular Center Hamburg, UKE Hamburg, Hamburg, Germany; 2 Department of Diagnostic and Interventional Radiology and Nuclear Medicine, University Medical Center Hamburg-Eppendorf, Hamburg, Germany; 3 German Center for Cardiovascular Research (DZHK), Partner Site Hamburg/Kiel/Lübeck, Hamburg, Germany; 4 Department of Cardiovascular Surgery, University Heart and Vascular Center Hamburg, UKE Hamburg, Hamburg, Germany; 5 Department of Vascular Medicine, German Aortic Center Hamburg, University Heart and Vascular Center Hamburg, UKE Hamburg, Hamburg, Germany

**Keywords:** Medical research, Epidemiology

## Abstract

Transthoracic echocardiography (TTE) and cine cardiac magnetic resonance imaging (CMR) are established imaging methods of the aortic root. We aimed to evaluate the comparability of measurements in TTE and standard cine CMR sequences of the aortic root. Our study included 741 subjects (mean age 63.5 ± 8 years, 43.7% female) from the Hamburg City Health Study (HCHS). Subjects underwent CMR and TTE. Aortic root measurements were performed at the level of the aortic annulus (AoAn), sinus of Valsalva (SoV), and sinotubular junction (STJ) by standard cine CMR in left ventricular long axis and left ventricular outflow tract view. Measurements were performed applying the leading-edge to leading-edge (LL) convention and inner-edge to inner-edge (II) convention in TTE and the II convention in CMR. Inter correlation coefficients (ICCs) demonstrated high inter- and intraobserver reproducibility for CMR and TTE measurements of SoV and STJ (ICCs 0.9–0.98) and moderate reproducibility for AoAn (ICCs 0.68–0.91). CMR measurements of SoV and STJ showed strong agreement with TTE: while correlations were comparable (r = 0.75–0.85) bias was lower with TTE II (bias − 0.1 to − 0.74) versus TTE LL measurements (mean bias − 1.49 to − 2.58 mm). The agreement for AoAn was fair (r = 0.51–0.57) with variable bias (mean bias 0.39–3.9). Standard cine CMR and TTE derived aortic root measurements are reproducible and comparable with higher agreement for TTE II instead of LL measurements. These results support an interchangeable application of TTE and standard CMR for screening of aortic root diseases thereby possibly reducing redundant multimodality imaging.

## Introduction

Dilatation of the aortic root is a frequent finding in clinical practice and is strongly associated with aortic regurgitation, an increased risk for aneurysm formation, and aortic dissection^1,2^. Timely diagnosis of aortic root dilatation is crucial because the abovementioned pathologies are associated with a high morbidity^3^ and mortality^4^.

Transthoracic echocardiography (TTE) is the most frequently used imaging method in the evaluation of the aortic root dimensions and most reference values for aortic diseases are derived from TTE studies^5^. However, cardiovascular magnetic resonance (CMR) has become increasingly available and allows for visualization and quantification of cardiac anatomy with accurate measurements of the aortic root^6^. It is performed in various clinical settings such as imaging of cardiomyopathies and in the setting of chest pain as it is part of major guideline recommendations^7^. CMR based aortic root diameter measurements overcome limitations of TTE such as a limited acoustic window and allow measurements perpendicular to the centreline of the aorta using multiplanar reconstructions as recommended in ESC guidelines^5,8^. Nevertheless, CMR is often used task-specific and not all possible sequences and angulations are performed. Previous studies report high agreement of specifically angulated CMR derived aortic root diameter measurements with TTE^9^. However, it is recommended to perform measurements of the aorta in a 3D dataset or sinus planes using a double-oblique orientation perpendicular to the aortic lumen^6,10^. Nevertheless, in clinical routine, specifically angulated planes or 3D sequences are only performed in patients with suspected aortic root disease. The cine CMR 3-chamber left ventricular (LV) view, which is part of the basic CMR sequences recommended by the Society for Cardiovascular Magnetic Resonance (SCMR) and the LV outflow tract (LVOT) view allow for a measurement of aortic root diameters as well^11^. However, there is a lack of data regarding the usefulness of these additional aortic root diameter measurements in the CMR 3-chamber LV and the LVOT cine-view in comparison to the established measurements performed by TTE.

Therefore, the purpose of this study was to provide data on the comparability of aortic root diameters obtained by cine-CMR and TTE in a large sample of the general population.

## Materials and methods

### Study population and study design

The Hamburg City Health Study (HCHS) was approved by the local ethics committee (PV5131, State of Hamburg Chamber of Medical Practitioners) and this study was approved by the review board (HCHS steering committee) of the HCHS. All participants gave written informed consent. This study and the HCHS were conducted in agreement with the *Declaration of Helsinki*.

The HCHS has been previously described^12^. In brief, the study prospectively includes a random sample of 45.000 participants between 45 and 74 years of age from the general population of Hamburg, Germany, investigating the interaction of socioeconomic risk factors, modern imaging techniques, physiological measurements, clinical variables, and targeted major diseases. At a baseline visit at the HCHS Epidemiological Study Center from the University Medical Center Hamburg all participants undergo a standardized interview, clinical examination, laboratory assessment, and TTE.

In this prospective study we analyzed a subgroup of the first 1000 HCHS participants who received a CMR^13^. Exclusion criteria were missing TTE data due to structural limitations at the beginning of the study (n = 209) or insufficient image quality in CMR and/or TTE (n = 57).

### Demographics and clinical parameters

Demographics and clinical parameters were investigated by standardised interviews and questionnaires conducted by medical professionals following standard operating procedures^12^. At the baseline visit at the HCHS Epidemiological Study Center blood samples were withdrawn under fasting conditions. BP was measured twice at the right upper arm in sitting position after 5 min of rest, results were averaged. Arterial hypertension was defined as systolic blood pressure ≥ 140 mmHg and diastolic blood pressure ≥ 90 mmHg, or the use of one or more of the following antihypertensive drugs: ACE inhibitors, angiotensin II receptor blockers, beta blockers, calcium channel blockers, renin inhibitors, or loop diuretics. Diabetes mellitus was determined by fasting glucose levels of ≥ 126 mg/dl, or the use of antidiabetic drugs. Coronary artery disease was self-reported by questionnaire and defined as having had a history from one or more of the following conditions: myocardial infarction, percutaneous coronary intervention (PCI) or coronary bypass surgery.

### Aortic root measurements

#### TTE

All TTE studies were evaluated and quantified at a single reading center blinded to the clinical information of the subjects using commercially available Siemens syngo SC2000 software (Siemens syngo SC 2000 Version 4.0, Siemens Healthineers, Erlangen, Germany). All TTE standard views were assessed in 2-dimensional echocardiography. The acquisition and analysis were performed according to the latest recommendations of the European Society of Cardiovascular Imaging (EACVI) and the American Society of Echocardiography (ASE)^14^.

**Figure 1:**
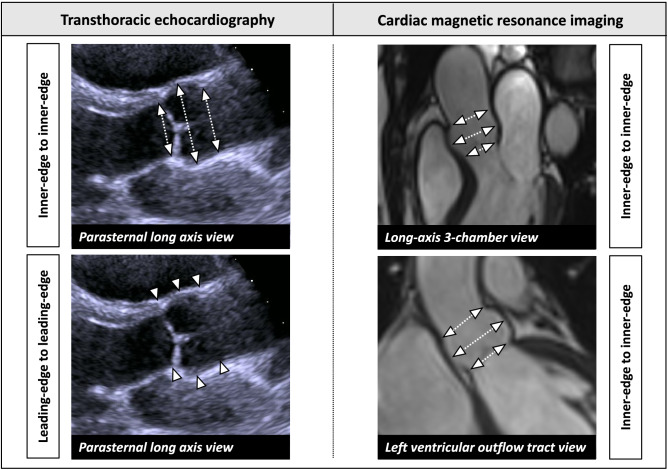
Measurements of the aortic root in TTE and CMR. Measurements were performed in end-diastole at the level of the aortic annulus, sinus of Valsalva and sinotubular junction. In TTE measurements were performed both from inner-edge to inner-edge (II) and from leading-edge to leading-edge. In CMR measurements were performed using the II convention.

The aortic root was assessed in transthoracic parasternal long axis view as recommended by the American Society of Echocardiography (ASE) and the European Association of Cardiovascular Imaging (EACVI) and previously described by our group^15,16^. Systematic measurements were performed perpendicular to the proximal aorta axis in end-diastole (ED) including the following: (a) aortic annulus (AoAn), (b) sinus of Valsalva (SoV), (c) sinotubular junction (STJ). Both for TTE and CMR, end-diastole was defined visually with a maximal distended left ventricle right before aortic valve opening. The AoAn was measured as the largest diameter between the hinge points of the right- and non-coronary cusps of the aortic valve. The SoV was measured as the maximum diameter of the aortic bulb. The STJ was assessed at the demarcated transition between the SoV and the tubular portion of the ascending aorta. All parts of the aortic root were measured perpendicular to the long axis of the aorta by the inner-edge to inner-edge (II) convention as well as the leading-edge to leading-edge (LL) convention (Fig. 1).

#### CMR

CMR was performed on 3 Tesla scanner (MAGNETOM Skyra, Siemens Healthineers, Erlangen, Germany). CMR protocols in the HCHS were described in detail in a previous publication^13^. balanced steady-state free precession imaging (BSSFP) cine-sequences were acquired in the following orientations: left ventricular (LV) short axis, 2,3,4-chamber LV long axis (LAX) view and left ventricular outflow tract (LVOT) view. Acquisition was performed standardized as recommended in the 2020 update for standardized cardiovascular magnetic resonance imaging (CMR) protocols published by the Society for Cardiovascular Magnetic Resonance (SCMR) Board of Trustees Task Force on Standardized Protocols^11^. BSSFP parameters were chosen as follows: voxel size 1.6 × 1.6 × 8 m^3^, FoV 340 mm^2^, TR 48 ms, TE 1.5 ms, FA 80°, parallel acquisition technique with a factor of 3.

All measurements were performed in the 3-chamber LV LAX cine-view and LVOT cine-view as they are part of standardized CMR protocols as recommended by the SCMR and comparable to the TTE PLAX^17^. Measurements were performed from the inner-edge to inner-edge at the predefined levels (Fig. 1): AoAn, SoV, and STJ. Diameter measurements were performed using a task specified plugin in ImageJ (v1.52, NIH, USA), which allowed for automatic export of diameter measurements.

##### Intra- and interobserver variability

For the calculation of intra- and interobserver agreement by interclass correlation coefficients (ICC [95%-CI]) a subset of 50 TTE and CMR exams was selected at random. For each imaging modality there were two individual readers resulting in a total of four different readers who exclusively measured CMR or TTE. Reader 1 performed two separate measurements (intraobserver variability) while reader 2 performed one measurement (interobserver variability) of all aortic root parts using the LL and II convention for TTE and II for CMR. For the prevention of recall bias there was a two-week interval between the reading sessions of reader 1.

### Statistical analysis

Statistical analyses were performed using R version 4.0.3. Continuous data are presented as median and inter-quartile-range (IQR). Categorical data are presented as absolute and relative frequencies. Missing values were not included in the calculation of frequencies.

The relationship between TTE and CMR variable was investigated by the Spearman’s correlation coefficient and Bland–Altman analysis (mean difference [95%-confidence interval (CI)]). A significance in the correlation was assumed if p-values were < 0.05.

### Declaration of Helsinki

The authors do hereby declare that their study complies with the Declaration of Helsinki.

## Results

### Study cohort

**Figure 2:**
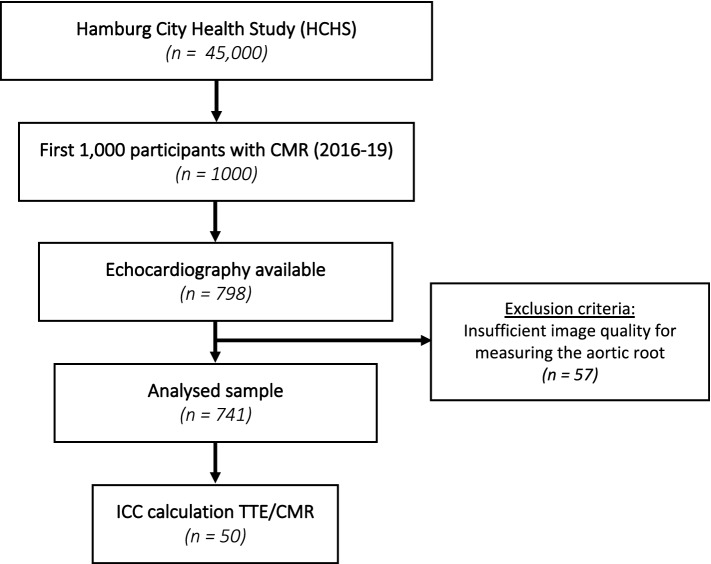
Study PRISMA. From the first 1000 HCHS subjects with CMR data, 798 subjects had undergone TTE examination, 57 were excluded due to insufficient echocardiographic or CMR image quality for measuring the aortic root. Inter and Intraobserver correlation coefficients were calculated for 50 at random selected subjects. *CMR* cardiac magnetic resonance imaging, *ICC* intra-/interclass correlation coefficient, *TTE* transthoracic echocardiography.

Among the first 1000 subjects with CMR enrolled into HCHS, 741 subjects were included in this analysis. The 741 subjects represented a middle-aged European population with 324 (43.7%) women, a median of 65 [IQR: 58–70] years, and a median body mass index (BMI) of 26.3 [IQR 23.8–29.4] kg/m^2^ (Fig. 2, Table 1). Hypertension was present in 516 (73.8%) subjects, 71 (10.4%) subjects had diabetes and 41 (8.3%) subjects coronary artery disease while atrial fibrillation and peripheral artery disease were present in 24 (3.3%) and 16 (2.3%) subjects, respectively. The median LDL-cholesterol level was 121 [IQR 98–143] mg/dl and the median TTE left ventricular ejection fraction 57.7 [IQR 54.9–61.5] %.

**Table 1:** Baseline characteristics of the study population.

	Males n = 417	Females n = 324
**Demographics**		
Age, years	66.0 [58.0, 70.0]	64.0 [57.0, 70.0]
BMI, kg/m^2^	26.7 [24.7, 29.7]	25.6 [22.8, 28.7]
Heart rate, bpm	68.0 [59.5, 76.0]	70.5 [63.5, 77.0]
Current smoker	71 (17.0)	70 (21.7)
**Comorbidities**		
Hypertension	306 (78.1)	210 (68.4)
Diabetes	48 (12.2)	23 (7.8)
Coronary artery disease	32 (10.8)	9 (4.5)
Atrial fibrillation	12 (2.9)	12 (3.7)
Peripheral artery disease	12 (3.2)	4 (1.3)
**Medication**		
Loop diuretics	4 (1.0)	4 (1.3)
Betablockers	69 (17.4)	58 (18.8)
ACEi/ARBs	155 (39.0)	106 (34.4)
**Laboratories**		
GFR, ml/min	83.5 [73.6, 90.9]	83.4 [72.7, 92.7]
NT-proBNP, ng/l	66.0 [39.0, 118.5]	110.5 [62.0, 182.8]
LDL-cholesterol, mg/dl	120.0 [94.0, 142.0]	124.0 [100.0, 146.0]
**Echocardiographic data**		
LVEF, %	57.1 [54.4, 60.1]	58.9 [55.9, 62.9]
TAPSE, mm	24.3 [21.5, 27.2]	23.7 [21.0, 26.4]
LV mass index, g/m^2^	92.7 [80.6, 107.9]	77.5 [68.9, 88.1]
LVEDV, ml	135.1 [116.2, 156.5]	102.4 [88.5, 115.6]

Continuous variables are presented as median and interquartile range, categorical variables are presented as absolute numbers and percentages.

*ACEi* angiotensin-converting enzyme inhibitor, *ARB* angiotensin receptor blocker, *BMI* body mass index, *GFR* glomerular filtration rate, *LAVI* left atrial volume index, *LDL* low-density lipoprotein, *LV* left ventricle, *LVEDV* left ventricular end-diastolic volume, *LVEF* left ventricular ejection fraction, *NT-proBNP* N-terminal pro-B-type natriuretic peptide, *TAPSE* tricuspid annular peak systolic excursion.

Median aortic root diameters in CMR LAX view were 21.3 [IQR 19.4, 23.5] mm for AoAn, 32.4 [29.8, 35.3] mm for SoV, and 26.6 [24.3, 29.4] mm for STJ, TTE and CMR LVOT diameters are presented in Table 2.

**Table 2:** Aortic root measured by transthoracic echocardiography and cardiac magnetic resonance imaging (CMR).

	TTE Inner-edge	TTE Leading-edge	CMR LAX view	CMR LVOT view
AoAn, mm	20.2 [18.7, 21.5]	21.1 [19.7, 22.4]	21.3 [19.4, 23.5]	23.9 [ 21.8, 28.5]
SoV, mm	32.6 [30.1, 35.5]	34.1 [31.4, 37.1]	32.4 [29.8, 35.3]	32.0 [29.2, 35.1]
STJ, mm	27.7 [25.5, 29.9]	29.7 [27.0, 31.9]	26.6 [24.3, 29.4]	26.7 [24.4, 28.9]

Continuous variables are presented as median and IQR.

*AoAn* aortic annulus, *CMR* cardiac magnetic resonance imaging, *LAX* long axis, *LL* leading-edge method, *LVOT* left ventricular outflow tract, *SoV* sinus of Valsalva, *STJ* sinotubular junction, *TTE* transthoracic echocardiography.

### Intra- and interobserver agreement of CMR and TTE measurements

ICCs of ≥ 0.9 demonstrated high inter- and intraobserver reproducibility for all CMR and TTE measurements of the SoV and STJ (Table 3). Generally, CMR showed higher ICCs than TTE. CMR LAX measurements showed higher ICCs than CMR LVOT measurements. Reproducibility of TTE LL and TTE II measurements did not differ significantly. Measurements of the SoV were both in CMR and TTE the most reproducible (SoV interobserver: TTE II ICC = 0.97 [0.95; 0.98]; TTE LL ICC = 0.94 [0.9; 0.97]; LAX ICC = 0.97 [0.95; 0.98]; LVOT ICC = 0.94 [0.9; 0.97].

**Table 3:** Inter- and intraobserver variability of aortic root measured by transthoracic echocardiography and cardiac magnetic resonance imaging measured by intra-class correlation coefficient.

	TTE Inner-edge	TTE Leading-edge	CMR LAX view	CMR LVOT view
**Interobserver correlation coefficient**				
AoAn	0.75 [0.6, 0.85]	0.68 [0.5, 0,8]	0.86 [0.76, 0.92]	0.8 [0.68, 0.88]
SoV	0.97 [0.95, 0.98]	0.94 [0.9, 0.97]	0.97 [0.95, 0.98]	0.94 [0.9, 0.97]
STJ	0.94 [0.9, 0.97]	0.92 [0.86, 0,95]	0.94 [0.9, 0.97]	0.9 [0.83, 0.94]
**Intraobserver correlation coefficient**				
AoAn	0.77 [0.62, 0.86]	0.82 [0.71, 0.91]	0.91 [0.84, 0.95]	0.78 [0.65, 0.87]
SoV	0.96 [0.93, 0.98]	0.95 [0.91, 0.97]	0.98 [0.97, 0.99]	0.94 [0.9, 0.97]
STJ	0.92 [0.86, 0.95]	0.92 [0.85, 0.95]	0.93 [0.89, 0.96]	0.93 [0.88, 0.96]

ICC is presented as mean and 95%-confidence interval.

Abbreviations as in Table 2.

In contrast, measurements of the AoAn showed lower intra- and interobserver reproducibility, indicated by lower ICCs both in TTE and CMR (AoAn interobserver: TTE LL ICC = 0.68 [0.5; 0.8], TTE II ICC = 0.75 [0.6; 0.85]; CMR LAX ICC = 0.86 [0.76; 0.92], CMR LVOT ICC = 0.8 [0.68; 0.88]).

### Comparison of CMR vs. TTE leading-edge aortic root measurements

**Figure 3:**
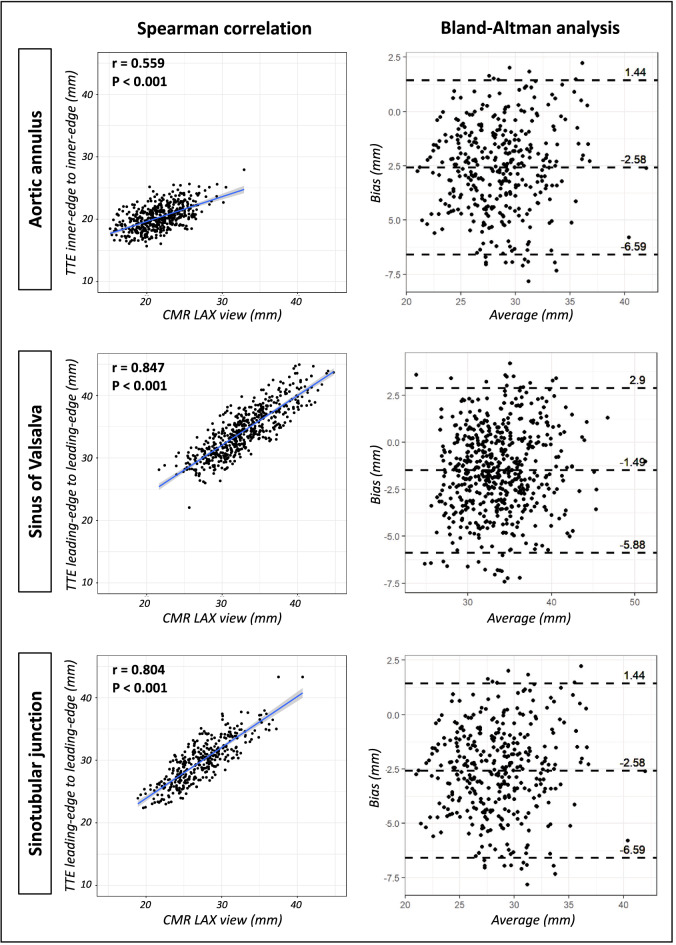
Comparison of TTE and CMR measurements of the aortic root. The following scatter plots show the linear regression line for two values as well as the spearman correlation coefficient and p-value. The Bland–Altman plots show the mean bias between the two values (CMR measurements subtracted by the corresponding TTE measurement) and the 95% confidence interval. *CMR* cardiac magnetic resonance imaging, *LAX* long-axis view, *TTE* transthoracic echocardiography.

CMR measurements of the SoV and STJ demonstrated strong correlations with TTE LL measurements (Table 4, Fig. 3, Supplemental Fig. 1). Concerning SoV diameters, the correlations with TTE LL varied depending on the CMR imaging plane: it was higher for the LAX view compared to the LVOT view (SoV TTE LL vs. CMR: LAX: r = 0.847, p < 0.001; LVOT: r = 0.746, p < 0.001). In contrast, correlations of the STJ with TTE LL diameters did not relevantly differ between LVOT or LAX view (STJ TTE LL vs. CMR: LAX: r = 0.804, p < 0.001; LVOT: r = 0.818, p < 0.001). CMR measurements generally resulted in smaller SoV and STJ diameters compared to TTE LL measurements (SoV TTE LL vs. CMR: LAX bias = − 1.49 ± 4.39 mm; LVOT bias = − 2.12 ± 5.79 mm; STJ TTE LL vs. CMR: LAX bias = − 2.54 ± 4.24 mm; LVOT bias = − 2.58 ± 4.01 mm).

**Table 4:** Spearman correlation and Bland–Altman analysis of TTE and CMR measurements.

	Spearman correlation		Bland–Altman analysis	
	Correlation coefficient	p-value	Bias (mean)	95% limits of agreement
*AoAn: TTE II vs. CMR LAX*	0.559	< 0.001	1.26	[− 3.46, 5.98]
*AoAn: TTE II vs. CMR LVOT*	0.514	< 0.001	3.9	[− 1.44, 9.24]
*AoAn: TTE LL vs. CMR LAX*	0.573	< 0.001	0.39	[− 4.27, 5.05]
*AoAn: TTE LL vs. CMR LVOT*	0.541	< 0.001	3.02	[− 2.2, 8.25]
*SoV: TTE II vs. CMR LAX*	0.846	< 0.001	− 0.1	[− 4.47, 4.26]
*SoV: TTE II vs. CMR LVOT*	0.751	< 0.001	− 0.71	[− 6.45, 5.03]
*SoV: TTE LL vs. CMR LAX*	0.847	< 0.001	− 1.49	[− 5.88, 2.9]
*SoV: TTE LL vs. CMR LVOT*	0.746	< 0.001	− 2.12	[− 7.91, 3.66]
*STJ: TTE II vs. CMR LAX*	0.807	< 0.001	− 0.7	[− 4.77, 3.37]
*STJ: TTE II vs. CMR LVOT*	0.807	< 0.001	− 0.74	[− 4.59, 3.1]
*STJ: TTE LL vs. CMR LAX*	0.804	< 0.001	− 2.54	[− 6.78, 1.69]
*STJ: TTE LL vs. CMR LVOT*	0.818	< 0.001	− 2.58	[− 6.59, 1.44]

*II* inner-edge to inner-edge, *LL* leading-edge to leading-edge. Other abbreviations as in Table 2.

Regarding the AoAn, CMR and TTE LL measurements showed fair agreement, regardless of the CMR imaging plane (AoAn TTE LL vs. CMR: LAX: r = 0.573, p < 0.001; LVOT: r = 0.541, p < 0.001). CMR measurements significantly overestimated the AoAn diameter compared with TTE LL measurements with moderate limits of agreement and a higher bias for the CMR LVOT view than for the LAX view (AoAn TTE LL vs. CMR: LAX bias = 0.39 ± 4.66 mm; LVOT bias = 3.02 ± 5.23 mm).

### Comparison of CMR vs. TTE inner-edge aortic root measurements

CMR measurements of the SoV and STJ, both in LAX and LVOT view, showed a high correlation with TTE II measurements (Table 4, Fig. 3, Supplemental Fig. 1). The mean difference of CMR vs. TTE II SoV and STJ measurements was lower compared to CMR vs. TTE LL measurements (SoV TTE II vs. CMR: LAX bias = − 0.1 ± 4.37; LVOT bias = − 0.71 ± 5.74 mm; STJ TTE II vs. CMR: LAX bias = − 0.7 ± 4.17; LVOT bias = − 0.74 ± 3.85 mm).

The correlation of AoAn CMR measurements with TTE II measurements was fair (AoAn TTE II vs. CMR: LAX: r = 0.559, p < 0.001; LVOT: r = 0.514, p < 0.001). CMR measurements overestimated AoAn dimensions compared to TTE II measurements. The overestimation was stronger with broader limits of agreement for the LVOT view (AoAn TTE II vs. CMR: LAX bias = 1.26 ± 4.72; LVOT bias = 3.9 ± 5.34).

## Discussion

Aortic root measurements in standardized cine CMR sequences are comparable to state-of-the-art TTE measurements in a contemporary, prospectively enrolled, middle-aged sample from the general population. The SoV and STJ showed a high degree of agreement, whereas it was only fair for the AoAn. Comparability improved if CMR measurements were compared to II instead of LL TTE measurements.

### Aortic root dimensions can be interchangeably measured by CMR or TTE

Few studies with only small cohorts compared aortic root measurements by TTE and CMR: In retrospective analyses with a maximum of 140 patients, diameters of the aortic root measured by CMR and TTE strongly correlated^18,19^. However, in those studies measurements were performed in specifically acquired sequences for measurements of the aortic root through the true cross sectional aortic valve plane^18^. To the best of our knowledge, there is only one retrospective study with a limited number of patients with suspected Marfan syndrome comparing standardized cine CMR measurements of the aortic root with TTE^17^. Hence our large prospective study in a general population is the first study to demonstrate that measurements assessed by LAX and LVOT cine CMR are comparable to TTE measurements. This is of clinical relevance as it supports not only the application of established TTE measurements but also of measurements derived from CMR orientations which are included in almost every CMR protocol for screening for aortic root diseases.

However, in CMR, the LAX view is part of routine clinical protocols whereas the LVOT view is not routinely included^8^. As LAX and LVOT view measurements for reliably assessing aortic root dimensions both demonstrated high correlations with TTE, the necessity for an additional LVOT view, at least regarding aortic root measurements, might be questionable.

Furthermore, the highest agreements were found for TTE according to LL convention with CMR rather than TTE II measurements and CMR^9^. This is in contrast to our results from our large cohort, which demonstrate comparable correlations, but a systematic overestimation of diameters measured by LL convention compared to CMR II measurements.

Diameters measured according to LL convention include the outer wall of one side of the aortic root. Therefore, an overestimation compared to diameters acquired by II convention, even though from different imaging modalities, seems comprehensible. Up to now, there is an ongoing discussion about which convention to use for echocardiographic measurements of the aortic root. While the ASE guidelines recommend to use the LL convention, the pediatric guidelines, supported by the 2010 American College of Cardiology and American Heart Association guidelines, support the II convention^14,20,21^. In clinical routine, as most reference values are based on the LL convention, TTE measurements are primarily performed using the LL convention^22^. However, in recent years technical upgrades and digital post-processing have led to major improvements in spatial resolution of transthoracic ultrasound. Thereby, the depiction of the thin aortic wall does not any longer limit the reproducibility of measurements^23^. Our results suggest a reconsideration of the LL-convention in TTE for improving comparability of SoV and STJ measurements with CMR.

Summarising, measurements of the SoV and AoAn highly correlated between TTE and CMR. Although we assessed a cohort with a low burden of aortic root dilatation, our data support an interchangeable clinical application of TTE and CMR for screening of incidental aortic root dilatation. However, CMR is still limited by lower availability, higher costs and more time-consuming examinations compared to TTE.

### CMR and TTE measurements of the aortic root are highly reproducible

Echocardiographic evaluation of the aortic root is a well-established method in clinical routine and recommended in patients with suspected aortic root disease^14,15^. Cine CMR is widely and increasingly adapted in routine clinical practice allowing for accurate measurements of the aortic root as well. However, data on reproducibility are derived from small cohorts or measurements limited to a single diameter of the ascending thoracic aorta^1,8^. In our population-based study, derived from 741 subjects, both methods demonstrated a very high reproducibility, with CMR even exceeding TTE, for measuring aortic root diameters especially for SoV and STJ.

### The complex structure of the AoAn limits reliable measurements and comparability

In contrast to measurements of SoV and STJ, diameters of the AoAn are routinely assessed according to II convention. However, both reproducibility and correlation of CMR and TTE measurements are reduced compared to measurements of SoV and STJ. The AoAn is an entity without a visible anatomic structure only virtually defined by the hinge-points of the three aortic valve leaflets^14,24^. This complex anatomy of the AoAn with an ellipsoid structure, in contrast to the more circular SoV and STJ, makes the exact measurement of the AoAn a challenging and error-prone process^25^. Only small alterations of the imaging plane, result in major differences of the measured diameter^8^. Individual alterations in dimension during the cardiac cycle further impair the reproducibility of AoAn assessment. Hence, our data suggest that measurements of the aortic annulus should only be performed in 3D CMR or computed tomography (CT) datasets with specifically angulated CMR planes.

### Limitations

As the study sample origins from the general population of Hamburg, most subjects are of Caucasian ascend and represent a predominantly healthy population. Hence, the translation of our findings into other populations, especially the typical patient collective in aortic root surgery, is limited.

Another limitation of this study is that 3D reconstructed CT scans, as the recommended standard of reference for the measurement of aortic root dimensions, were not performed^5^. No conclusions can be drawn about the accuracy of each the modalities. Nevertheless, the aim of our study was to investigate whether measurements of the aortic root in standard planes of a regular cine-CMR are comparable to state-of-the-art TTE measurements. By showing the high correlation of both imaging techniques, these additional CMR measurements show high validity when compared to TTE and could be implemented in clinical routine for screening purposes and prevent or trigger further imaging.

The ascending aorta was not measured, hence no conclusions can be drawn regarding dilatations above the aortic root. Two further limitations have to be considered: First, since no blood pressure measurements were performed during TTE and CMR examinations, a possible impact of blood pressure fluctuations on aortic root dimensions, e.g. a raised blood pressure during CMR examination, was not considered. Second, TTE and CMR examinations were not performed on the same day. However, the median time interval between the two examinations was only 28 days. Consequently, it is highly unlikely that during this short period, aortic root diameters significantly changed.

## Conclusion

In this study, derived from a large sample of the general population, CMR measurements of the aortic root were comparable to standard of care TTE measurements. Reproducibility and agreement were excellent for the SoV and STJ, while they were only fair for the AoAn. Comparability improved if the II instead of the LL convention was used, challenging the established LL-convention in aortic root echocardiography. These results suggest that cine CMR and TTE might be applied interchangeably in clinical routine thereby possibly reducing redundant multimodality imaging.

## Supplementary Information

Supplementary Figure 1.

## Data Availability

The data underlying this article cannot be shared publicly due to the privacy of individuals that participated in the study. The data will be shared on reasonable request to the corresponding author.

## Abbreviations

AF: Atrial fibrillation
AoAn: Aortic annulus
ASE: American Society of Echocardiography
BMI: Body mass index
CMR: Cardiac magnetic resonance imaging
GFR: Glomerular filtration rate
EACVI: European Association of Cardiovascular Imaging
ESC: European Society of Cardiology
HCHS: Hamburg City Health Study
LAVI: Left atrial systolic volume indexed to body surface area
LAX: Long axis
LVEF: Left ventricular ejection fraction
LVMI: Left ventricular mass index
LVOT: Left ventricular outflow tract
NT-proBNP: N-terminal pro-B-type natriuretic peptide
SCMR: Society for Cardiovascular Magnetic Resonance
SoV: Sinus of Valsalva
STJ: Sinotubular junction
TR Vmax: Maximal tricuspid regurgitation velocity
TTE: Transthoracic echocardiography
