# Supplementary material for: Comparison of cine cardiac magnetic resonance and echocardiography derived diameters of the aortic root in a large population-based cohort
Source: Sci Rep. 2022 Sep 12;12:15307. doi: 10.1038/s41598-022-19461-5 (PMC9468025; doi:10.1038/s41598-022-19461-5)
Supplement: Supplementary file 1 — Supplementary Figure 1. [file 41598_2022_19461_MOESM1_ESM.docx]

**Supplements**

**Supplemental Figure 1. Comparison of TTE inner-edge to inner-edge and CMR measurements of the sinus of Valsalva and the sinotubular junction.**

The following scatter plots show the linear regression line for two values as well as the spearman correlation coefficient and p-value. The Bland-Altman plots show the mean difference (bias) between the two values and the 95% confidence interval. Abbreviations: CMR = cardiac magnetic resonance imaging, LAX = long-axis view, TTE = transthoracic echocardiography.
